# Exploring oxidative stress and epigenetic biomarkers in bees: insights into health effects of air pollution and diet – a pilot study

**DOI:** 10.1007/s11356-026-38040-z

**Published:** 2026-07-07

**Authors:** Roberta Giorgione, Marcello Messi, Daniela Pigini, Maria Luisa Astolfi

**Affiliations:** 1 Department of Chemistry, Sapienza University of Rome, P.Le Aldo Moro 5, 00185 Rome, Italy; 2 Department of Medicine, Epidemiology, Environmental and Occupational Hygiene, INAIL, Via Fontana Candida 1, 00078 Monte Porzio Catone, Italy; 3 Research Center for Applied Sciences to the Safeguard of Environment and Cultural Heritage (CIABC), Sapienza University of Rome, P.Le Aldo Moro 5, 00185 Rome, Italy

**Keywords:** Biomonitoring, Environmental pollution, HPLC–MS/MS, Nucleic acid oxidation, Oxidative stress, Pollinators

## Abstract

Bees are vulnerable to environmental pollution, including toxic emissions from fire events, which can impact oxidative balance. This study was conducted as a pilot investigation with a relatively limited sample size and aimed to assess the levels of specific biomarkers [8-oxo-7,8-dihydroguanine (8-oxoGua), 8-oxo-7,8-dihydroguanosine (8-oxoGuo), 8-oxo-2′-deoxyguanosine (8-oxodGuo), 3-nitrotyrosine (3-NO₂Tyr), and 5-methylcytidine (5-MeCyt)] by HPLC‒MS/MS analysis in bees sampled before (May) and after (June) a landfill fire. The bees were divided into three groups: untreated (control), supplemented with *Quassia amara*, or fed probiotics. HPLC‒MS/MS analysis revealed no significant differences in the total bee population before and after fire. However, 8-oxoGua significantly increased in the control bees postfire (p < 0.05). Conversely, bees treated with *Q. amara* or probiotics tended to exhibit lower levels of oxidative and epigenetic biomarkers. However, only the *Q. amara* group showed a significant post-fire decrease in 8-oxoGua (p < 0.010). These observations should be considered preliminary and hypothesis-generating given the pilot nature and limited sample size of the study. Further investigations are required to elucidate their interaction mechanisms and long-term effects on pollinator health. Overall, this study highlights the potential of honeybees as early sentinels of oxidative stress, providing valuable insights for environmental monitoring and ecosystem health assessment.

**Supplementary Information:**

The online version contains supplementary material available at 10.1007/s11356-026-38040-z.

## Introduction

Bees are indispensable pollinators that play a pivotal role in maintaining biodiversity and supporting agricultural productivity (Gekiére et al. 2023; Murphy et al. 2022; Potts et al. 2016). However, recent decades have witnessed alarming declines in bee populations globally, attributed to various environmental stressors, including pesticide exposure, habitat loss, and environmental pollution (Brunet and Fragoso 2024). An emerging environmental concern is the occurrence of fires in waste landfills, which can release a complex mixture of toxic substances into the environment (Giampaoli et al. 2025). Owing to their foraging behavior and close contact with environmental matrices, bees are particularly susceptible to pollutants released during landfill fires (Giampaoli et al. 2025). Inhalation of contaminated air, ingestion of polluted nectar and water, and contact with contaminated surfaces can lead to the accumulation of toxic substances in their bodies (Astolfi et al. 2022; Badiou-Bénéteau et al. 2012; Carvalho et al. 2013; Gekiére et al. 2023; La Porta et al. 2023). Honey bees are increasingly recognized as effective bioindicator organisms in environmental monitoring due to their wide foraging range, high sensitivity to environmental contaminants, and ability to integrate exposure across different environmental compartments. Recent studies have demonstrated their applicability in monitoring atmospheric pollutants, trace elements, volatile organic compounds, and soil-derived contaminants, as well as in evaluating environmental quality through analyses of bees and bee-derived products (Ilić et al. 2024; Mair et al. 2023; Marcoccia et al. 2024; Praus et al. 2024; Trimmel et al. 2026). These findings further support their role as complementary bioindicator systems for assessing anthropogenic impacts on ecosystems.

One critical yet underexplored factor contributing to bee health deterioration is oxidative stress, a state characterized by an imbalance between the production of reactive oxygen species (ROS) and the organism's antioxidant defenses, leading to cellular and molecular damage (Biová et al. 2025; Liguori et al. 2018; Messi et al. 2025). Oxidative stress can result in significant damage to nucleic acids, proteins, and lipids, impairing their cellular functions and viability (Fichman et al. 2023; Gammella et al. 2016; Kohen and Nyska 2002; Messi et al. 2025). Biomarkers are valuable tools for evaluating the health of bioindicator organisms, providing crucial insights into environmental quality by reflecting the effects of anthropogenic stressors at various biological levels across different bee species (Badiou-Bénéteau et al. 2012; Carvalho et al. 2013; Caliani et al. 2021; Lupi et al. 2021; La Porta et al. 2023). Frequently employed biomarkers include enzymatic activity measurements, such as those of cholinesterase (ChE) for assessing neurotoxicity, catalase (CAT) for the antioxidant response, glutathione S-transferase (GST) for detoxification, and lipid peroxidation (LPO) levels for evaluating oxidative stress (Badiou-Bénéteau et al. 2012; Biová et al. 2025; Lupi et al. 2021; Messi et al. 2025).

Biomarkers have been instrumental in assessing the impact of various environmental stressors on bee populations, including exposure to urban and industrial pollution (Nikolić et al. 2016; Al Naggar et al. 2020; Li et al. 2024) as well as pesticides (Gauthier et al. 2018; Tan et al. 2022; Benito-Murcia et al. 2024; Ayoub et al. 2024; Hisamoto et al. 2024; Mackei et al. 2024). A recent study by Giampaoli et al. (2025) investigated the consequences of a landfill fire at the Malagrotta site in Rome, Italy, on bee health. The research has focused on assessing the physiological stress and oxidative protein damage caused by exposure to environmental pollutants by measuring H_2_O_2_ levels and protein carbonyl content, respectively. The findings revealed that pollutants from landfill fires increased oxidative stress and metal accumulation in bees and support the role of bees as bioindicator models for studying biomarkers associated with environmental contamination (Badiou-Bénéteau et al. 2012; Carvalho et al. 2013; La Porta et al. 2023). To improve assessments of anthropogenic effects on bee populations, it is essential to adopt an integrated approach that combines classical enzymatic and oxidative markers with biomarkers reflecting oxidative and epigenetic damage to nucleic acids and proteins. This strategy allows a more accurate evaluation of environmental stress exposure (Badiou-Bénéteau et al. 2012; Lupi et al. 2021). In humans, exposure to environmental contaminants has been linked to oxidative modifications of nucleic acids, including guanine oxidation products such as 8-oxo-7,8-dihydroguanine (8-oxoGua), 8-oxo-7,8-dihydroguanosine (8-oxoGuo), and 8-oxo-7,8-dihydro-2′-deoxyguanosine (8-oxodGuo) (Chao et al. 2021; Domingo-Relloso et al. 2019; Tanaka and Chock 2021). Oxidative modifications also affect proteins and lipids; for example, tyrosine nitration produces 3-nitrotyrosine (3-NO₂Tyr), a widely recognized biomarker of protein oxidation (Kehm et al. 2021), while alterations in 5-methylcytidine (5-MeCyt), a methylation product of cytidine and an epigenetic marker of RNA turnover, are associated with disease-related biological processes (Buonaurio et al. 2021; Guo et al. 2018). This study applies the same panel of oxidative stress and epigenetic biomarkers (8-oxoGua, 8-oxoGuo, 8-oxodGuo, 3-NO₂Tyr, and 5-MeCyt) to honeybees to evaluate the effects of environmental contaminants. These markers were selected because they are widely used in human biomonitoring to assess exposure-related oxidative damage and biological responses (Buonaurio et al. 2021; Giorgione et al. 2025). To our knowledge, no previous studies have applied this comprehensive panel to bees, highlighting their potential as early sentinels of oxidative stress, which may provide insight into environmental health risk assessment frameworks. Given the complexity of real-world exposure scenarios and the difficulty in identifying suitable control apiaries unaffected by local pollution sources, the present investigation should be regarded as a pilot study and the interpretation of biomarker responses should consider potential temporal and environmental confounding factors.

Collectively, recent advances in honey bee biomonitoring demonstrate a shift from single-matrix contamination assessment toward integrated multi-matrix and multi-stressor environmental evaluation. However, most existing studies have focused primarily on chemical or elemental accumulation, while fewer have addressed early molecular effects of exposure at the level of oxidative damage and nucleic acid modifications. The present study addresses this gap by integrating a comprehensive panel of oxidative stress and epigenetic biomarkers in honey bees, thereby extending their application from exposure monitoring to early biological effect assessment and improving the mechanistic interpretation of environmental stress responses.

## Materials and methods

### Study area and sample collection

As previously described (Giampaoli et al. 2025), the sampling site was located near the Malagrotta landfill in the province of Rome, central Italy. Briefly, six hives of *Apis mellifera ligustica Spinola*, with similar colony sizes and bee populations, were selected. Two hives served as controls and received only a control solution (1 L; Candiplus1; Zucchero and C., Florence, Italy). The four remaining hives, which formed the experimental groups, were divided into two pairs: one pair was provided with a sugar solution (1 L) containing bee-specific probiotics (10 g; Probee; CHRI. VA, Rome, Italy), while the other received a sugar solution (1 L) supplemented with 5% *Quassia amara* (Bitterholz, Quassiaholz gemahlen, Naturix24, Germany) (Astolfi et al. 2022; Giampaoli et al. 2025). During sampling (April–July 2022), a major fire broke out at a mechanical–biological waste treatment plant located approximately 500 m from the sampling area. This fire released a cloud of smoke over the region and persisted for several days due to the combustion of highly flammable materials, leading to the emission of numerous contaminants.

No external control apiary was included in the study design. The identification of a suitable control site was considered impracticable because an ideal control apiary should be located within the same geographical area and experience comparable environmental and climatic conditions while remaining unaffected by both local pollution sources and emissions associated with the landfill fire. Consequently, comparisons were performed using samples collected before and after the fire event within the same apiary. We acknowledge that this approach cannot completely disentangle the potential effects of the fire from seasonal variation and natural changes in colony dynamics.

For oxidative stress analysis, approximately 30 bees per hive were collected from each hive and processed separately per colony. Each hive sample was placed in an independent 50-mL Falcon® tubes (Corning Incorporated Life Sciences, Amsterdam, The Netherlands), ensuring that samples were not pooled across hives. The collected bees were immediately flash-frozen in liquid nitrogen (~ 196 °C). These samples were then transported to the laboratory and stored at −80 °C until further processing.

### Chemicals and reagents

The analytical reference standards for 8-oxoGua, 8-oxoGuo, and 8-oxodGuo were obtained from Spectra 2000 SRL (Rome Italy). The isotope-labeled internal standard (^13^C^15^N₂) 8-oxoGua (98%) was also sourced from Cambridge Isotope Laboratories (Tewksbury, MA, United States), whereas (^13^C^15^N₂) 8-oxoGuo, (^13^C^15^N₂) 8-oxodGuo, and cotinine-d_3_ (99.9%) were supplied by CDN Isotopes Inc. (Pointe-Claire, QC, Canada). The 3-NO₂Tyr standard was provided by Cayman Chemical Company (Ann Arbor, MI, USA), whereas 3-NO₂Tyr d₃ was obtained from Toronto Research Chemicals (Toronto, ON, Canada). 5-MeCyt (≥ 99%) was supplied by Sigma‒Aldrich, Merck (St. Louis, MO, USA). The additional chemicals, including glacial acetic acid, 30% NH₃, dimethyl sulfoxide (DMSO), sodium hydroxide solution (50–52% in water), Chromasolv® gradient-grade methanol (99.9%) and acetonitrile for HPLC/MS (99.9%), as well as low benzene content carbon disulfide, were acquired from Sigma‒Aldrich (Saint Louis, MO, USA). Purified water was generated via a Milli‒Q Plus system (Millipore, Milford, MA, USA). Anotop 10LC syringe filter devices (0.2 µm pore size, 10 mm diameter) were sourced from Whatman Inc. (Maidstone, UK).

### Bee sample preparation

Individual bee samples (~ 150 mg fresh weight) were thawed at room temperature and extracted in a mixture consisting of 300 µL of water and 600 µL of methanol. After centrifugation at 11,000 rpm for 10 min, the supernatant was transferred to a centrifugal filter unit (0.2 μm pore size, 10 mm diameter; Whatman Inc., Maidstone, UK). Before HPLC‒tandem mass spectrometry (HPLC‒MS/MS) analysis, a 100 µL aliquot of the filtered sample was mixed with the internal standard solution at a ratio of 1:3 and vortexed for 1 min. For each hive, between 3 and 6 individual bees were analyzed depending on sample availability. Overall, eight individual bees were analyzed for each treatment group (control, probiotics, and *Q. amara*) in each sampling month, corresponding to 24 bees per month and 48 bees overall.

### HPLC‒MS/MS analysis

The concentrations of 8-oxoGua, 8-oxoGuo, 8-oxodGuo, and 3-NO_2_Tyr in bee samples were determined by HPLC–MS/MS according to a previously described method (Andreoli et al. 2010), with modifications reported in Giorgione et al. (2025). The system consisted of an API 4000 triple-quadrupole mass spectrometer equipped with a Turbo Ion Spray (TIS) probe (AB Sciex, Framingham, MA, USA) coupled to a Series 200 LC quaternary pump (PerkinElmer, Norwalk, CT, USA). Chromatographic separation was performed on a Kinetex Polar C18 100 Å column (150 × 4.6 mm, 2.6 μm, Phenomenex, Castelmaggiore, Italy) using a gradient mixture of acetonitrile and methanol (9:1 v/v) with 0.5% acetic acid in water (all reagents from Carlo Erba Reagents, Milan, Italy) at a flow rate of 500 µL/min. The injection volume was 20 µL, and ionic transitions (precursor → product) were monitored in positive mode. Instrument control and data analysis were performed using Analyst® software (version 1.5, AB/Sciex, Concorde, ON, Canada). Table 1 reports the ionic transition retention times of 8-oxoGua, 8-oxoGuo, 8-oxodGuo, 3-NO₂Tyr, and 5-MeCyt, along with the corresponding internal standards.

**Table 1:** Ionic transitions and retention times of analytes and internal standards

Compounds	Ionic transitions	Retention time
(8-oxoGua)	168.402 → 140.100	4.46
(^13^C^15^N_2_) 8-oxoGua	171.139 → 142.100	
8-oxoGuo	300.268 → 168.300	5.45
(^13^C^15^N_2_) 8-oxoGuo	303.240 → 171.000	
8-oxodGuo	284.000 → 168.100	5.7
(^13^C^15^N_2_) 8-oxodGuo	287.163 → 171.100	
3-NO_2_Tyr	226.992 → 181.000	5.99
3-NO_2_Tyr-d_3_	229.992 → 184.000	
5-MeCyt	257.946 → 126.100	3.94
Cotinine-d_3_	180.300 → 80.100	

Before injection, extracted bee samples were vortexed and mixed with the internal standard. Each sample was analyzed in duplicate, and the arithmetic mean of the peak areas of the two replicates was used for quantification.

Calibration curves (Fig. S1) were prepared by stepwise dilution of stock standard solutions in methanol. Concentration ranges were: 0–900 µg/L for 8-oxoGua and 3-NO₂Tyr, 0–75 µg/L for 8-oxoGuo, 0–23 µg/L for 8-oxodGuo, and 0–64 µg/L for 5-MeCyt. Internal standard stock solutions were added to each calibration standard at the following concentrations: 400 µg/L for (^13^C^15^N₂) 8-oxoGua, 50 µg/L for (^13^C^15^N₂) 8-oxoGuo, 15 µg/L for (^13^C^15^N₂) 8-oxodGuo, 340 µg/L for 3-NO₂Tyr-d₃, and 375 µg/L for cotinine-d₃. Quantification was performed using the internal standard method. Fig. S1 shows the calibration curves for all biomarkers.

Linearity was assessed by correlating the ratio of the analyte peak area to that of its internal standard against analyte concentration, with R^2^ > 0.99 considered satisfactory. The limit of detection (LOD) was defined as the lowest concentration with a signal-to-noise ratio (SNR) of 3, and the limit of quantification (LOQ) was defined as the concentration with an SNR of 10. LOD and LOQ values for each biomarker were as follows: 8-oxoGua, 0.003 and 0.01 mg/kg; 8-oxodGuo, 0.0008 and 0.003 mg/kg; 8-oxoGuo, 0.004 and 0.01 mg/kg; 3-NO₂Tyr, 0.01 and 0.04 mg/kg; 5-MeCyt, 0.002 and 0.006 mg/kg; cotinine, 0.09 and 0.2 mg/kg. These parameters demonstrate the sensitivity and suitability of the method for accurate biomarker quantification in bee samples.

### Statistics

Statistical analyses were conducted using the IBM SPSS statistics 27 software (IBM Corp., Armonk, NY, USA). Initially, the distribution of each analyte concentration was assessed via both the Kolmogorov–Smirnov and Shapiro–Wilk normality tests (Faraway 2004). Since the data did not follow a normal distribution, a log-normal transformation was applied. Once normality was confirmed, the transformed data were analyzed by one-way ANOVA, followed by Bonferroni post hoc correction to detect statistically significant differences among the concentrations. It should be noted that multiple bees were sampled within the same hive; therefore, individual bees may not represent fully independent statistical units. This limitation was considered when interpreting the results, and statistical inference should be regarded as exploratory at the hive level.

Focused principal component analysis (FPCA) was carried out following the method described by Falissard (1999), using a custom MATLAB function (R2020b, MathWorks, Portola Valley, CA, USA) based on Spearman’s rank correlation. The analysis was performed on combined datasets integrating oxidative stress biomarkers (8-oxoGua, 8-oxoGuo, 8-oxodGuo, 3-NO₂Tyr, and 5-MeCyt) with indicators of physiological stress (PS) and potential post-transcriptional damage (PTD) in bees, the latter being discussed and reported in Giampaoli et al. (2025).

A p value of less than 0.05 was considered indicative of statistical significance for all tests.

## Results and discussion

**Fig. 1:**
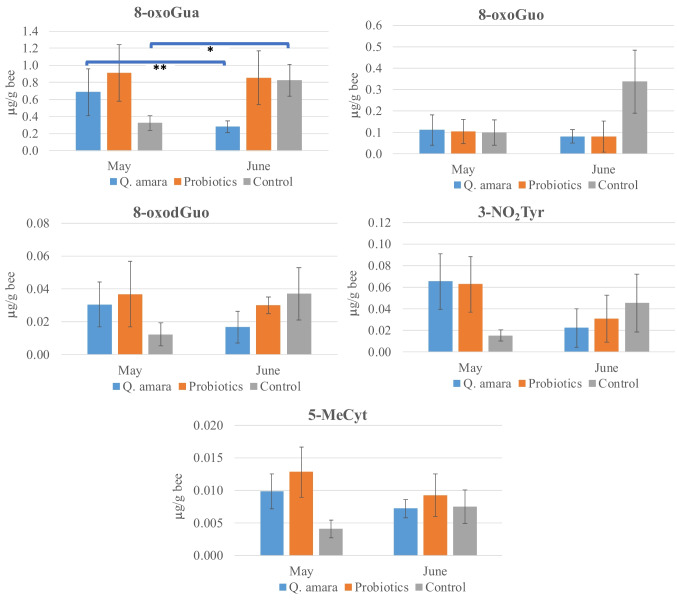
Concentration trends (µg/g bee) of oxidative stress biomarkers in honey bees. Biomarker levels were determined in bees fed with control solution, *Q. amara*, or probiotics before (May) and after (June) the fire event. Each treatment group comprised two independent hives, with 3–5 individual bees analyzed per hive depending on sample availability. Bars represent the arithmetic mean concentrations of the analyzed bees, and error bars indicate the standard deviation (SD). Significance levels: * = *p* < 0.05; ** =*p* < 0.01

The results of the HPLC‒MS/MS analysis are shown in Fig. 1 and Table 2. When the entire bee population was considered, no statistically significant differences (p > 0.05) in oxidative stress or epigenetic biomarker levels were detected before (May) and after (June) the fire event (Table 2). This suggests that, when averaged across all individuals, the potential effects of the fire event or of differences in diet were likely diluted by the natural variability among colonies. However, when the data were analyzed by treatment (Fig. 1), distinct trends became apparent. In particular, the control group exhibited a general increase in oxidative and epigenetic biomarker concentrations following the fire. These results indicate that averaging data across the entire population may obscure treatment- or colony-specific patterns and should therefore be interpreted only as a descriptive overview. In particular, the mean concentration of 8-oxoGua in control bees was significantly greater in June than in May (*p* < 0.05). Conversely, bees fed with *Q. amara* or probiotics tended to exhibit lower concentrations of several biomarkers after the fire event. However, only the *Q. amara* group showed a significant post-fire decrease in 8-oxoGua levels (*p* < 0.010).

**Table 2:** Oxidative biomarker levels (µg/g bee) in the entire bee population measured before (May) and after (June) the fire event

	8-oxoGua	8-oxoGuo	8-oxodGuo	3-NO_2_Tyr	5-MeCyt
May					
N^a^	24	24	24	24	24
Mean	0.59	0.105	0.024	0.045	0.0081
SD	0.32	0.059	0.018	0.039	0.0043
Median	0.60	0.095	0.022	0.026	0.0084
Min	0.18	0.040	0.007	0.001	0.0029
Max	1.28	0.234	0.070	0.121	0.0167
June					
N^a^	24	24	24	24	24
Mean	0.56	0.17	0.026	0.031	0.0076
SD	0.36	0.21	0.018	0.038	0.0021
Median	0.39	0.10	0.027	0.021	0.0074
Min	0.17	0.03	0.007	0.003	0.0049
Max	1.29	0.81	0.075	0.130	0.0116
df_total_^b^	47	47	47	47	47
F (1,46)^b^	0.132	0.363	0.103	1.27	0.074
p^b^	0.720	0.552	0.751	0.270	0.788
η^2b^	0.005	0.014	0.004	0.048	0.003

^a^N represents the number of individual bee samples analyzed for each biomarker in each sampling period

^b^Separate one-way ANOVA analyses were performed for each biomarker to compare values between May and June in the overall bee population; the corresponding F statistics, exact p values, and effect sizes (η^2^) are reported for each biomarker. The ANOVA yielded F(1,46), where 1 and 46 represent the between-group and within-group degrees of freedom, respectively, corresponding to a total of 47 degrees of freedom (df_total = 47)

The results obtained in this study suggest a potential association between environmental pollution—particularly waste fires— and oxidative stress responses in bees, primarily reflected by changes in 8-oxoGua levels. In that study, acute exposure to fumes from waste combustion at the Malagrotta landfill led to an increase in hydrogen peroxide and protein carbonyl levels in untreated bees, indicative of physiological stress and oxidative protein damage caused by exposure to environmental pollutants. In the present study, a significant increase in 8-oxoGua was observed in control bees following the pollution event (p < 0.05). 8-oxoGua can originate from both RNA and DNA oxidation (Hahm et al. 2022), and this increase may reflect a response to airborne contaminants, consistent with the molecular damage at the protein level reported by Giampaoli et al. (2025). As shown in Fig. S2, physiological stress (PS) displayed a significant positive correlation with 8-oxoGuo, suggesting a link between systemic stress and RNA oxidation. However, this relationship should be interpreted cautiously, as it was not consistently observed across all analyzed biomarkers.

Conversely, no significant correlations were observed between the potential post-transcriptional damage (PTD) identified by Giampaoli et al. (2025) and the oxidative stress biomarkers analyzed in the present study (Fig. S3). This lack of association suggests that PTD and the nucleic acid oxidation markers measured here may reflect distinct but complementary stages of the cellular stress response; however, the limited consistency across biomarkers does not support broader generalization regarding systemic or epigenetic-level regulation.

These findings highlight the potential harmful effects of acute pollution events, such as waste fires, which can release a broad spectrum of contaminants—including heavy metals (As, Cd, Cr, Cu, Hg, Ni, Pb, Se, and Zn), volatile and persistent organic compounds, aldehydes, and fine particulate matter (Lemieux 1997; EEA 2016; Bihałowicz et al. 2021). However, it should be noted that this was an accidental and unpredictable event, which made it impossible to perform simultaneous sampling in a truly uncontaminated area. Moreover, identifying an appropriate distance for comparison is inherently complex, as moving further away from the affected site could introduce additional variability due to differences in foraging activity, local environmental conditions, and background pollution levels. Therefore, while the results suggest a potential association between the fire event and oxidative stress responses, they should still be interpreted with caution and not as evidence of systemic or regulatory-level biological effects.

The present data suggest a potential modulation of oxidative stress biomarkers in bees associated with probiotic supplementation and *Q. amara* treatment. These observations are consistent with literature demonstrating that probiotics can modulate the gut microbiota of bees, enhancing tolerance to chemical agents and preventing dysbiosis (Gekiére et al. 2023; Wu et al. 2020; Wang et al. 2021), whereas phenolic compounds from *Q. amara* may exert antioxidant activity via free radical scavenging (Manach et al. 2004; Zargoosh et al. 2019). Previous studies also support this trend: in Maccarese, a background area, probiotic supplementation reduced concentrations of some contaminants in bees (Astolfi et al. 2022), a trend later confirmed in bees exposed to pollutants at Malagrotta (Giampaoli et al. 2025).

Overall, exposure to pollutants released by waste fires represents a potential risk factor for oxidative stress in bees. Nonetheless, the data indicate that medicinal plants and probiotic bacteria may contribute to modulating oxidative stress responses in bees rather than exerting direct protective effects, potentially supporting pollinator resilience under environmental stress conditions. However, additional studies are warranted to investigate the factors that may influence the adsorption capacity of these bioadsorbents and their excretion pathways in bees. This is particularly relevant given that bees and other pollinators are exposed to a complex mixture of chemical pollutants in the environment, often at variable concentrations, which may lead to antagonistic, additive, or synergistic effects (Monchanin et al. 2021a, b; Gekiére et al. 2023).

## Study limitations and future directions

It should be noted that bees collected from the same hive may not be statistically independent, as they share similar environmental conditions, diet, and exposure patterns. This potential lack of statistical independence, which is common in field-based studies, may slightly reduce the statistical power and generalizability of the findings. An additional limitation of the present study is the potential pseudoreplication arising from the sampling of multiple bees within the same hive. Because bees from the same colony are not fully independent observations and the number of hives per treatment was limited, the statistical inference should be interpreted with caution and the findings should be considered preliminary and exploratory, consistent with the pilot nature of the study. Moreover, the limited number of independent hives per treatment did not allow a robust evaluation of treatment × sampling-period interactions; therefore, any apparent treatment-related differences should be regarded as exploratory and hypothesis-generating rather than as evidence of a protective effect. Nevertheless, the adopted sampling strategy allowed for meaningful preliminary insights into the effects of environmental stressors on oxidative and epigenetic biomarkers in bees.

Future studies could strengthen the experimental design by increasing the number of hives, incorporating reference colonies located in areas with lower expected pollution levels, and expanding the geographic and temporal scope of sampling. However, identifying truly uncontaminated environments and ensuring comparable foraging conditions across colonies remain challenging tasks. Moreover, integrating complementary approaches—such as multi-omics analyses or controlled exposure experiments—would enable a more comprehensive understanding of the molecular mechanisms underlying biomarker responses and enhance the robustness and applicability of the results. Future studies including a larger number of independent colonies are therefore required to provide more robust estimates of treatment effects, to formally assess treatment × sampling-period interactions, and to confirm the exploratory findings reported here.

## Conclusion

Bees are vital components of ecosystems, and their decline poses significant threats to biodiversity and food security. Environmental pollution, including emissions from landfill fires, may be associated with oxidative stress responses in bees, potentially leading to adverse health outcomes. Monitoring oxidative stress and epigenetic biomarkers such as 8-oxoGua, 8-oxoGuo, 8-oxodGuo, 3-NO₂Tyr, and 5-MeCyt provides valuable insights into the physiological status of bees under environmental stress; however, the present results indicate that only 8-oxoGua showed a consistent and statistically significant response, while the other biomarkers displayed more variable or non-significant changes. The use of advanced analytical techniques such as HPLC–MS/MS facilitates accurate biomarker detection, supporting comprehensive assessments of bee health.

However, it should be emphasized that oxidative stress in bees may result from a combination of factors, including diet, environmental contaminants, seasonal variation, forage availability, meteorological conditions, and natural colony dynamics, rather than being solely attributable to specific pollution events such as landfill fires. Although comparison with colonies from cleaner background areas would ideally strengthen the interpretation of the results, identifying a truly suitable control apiary is challenging, as it should share similar geographical, climatic, and ecological characteristics with the study area while remaining unaffected by local pollution sources and by emissions associated with the landfill fire. Moreover, ensuring that bees from different colonies forage on similar floral resources and are exposed to comparable environmental conditions remains difficult. Therefore, the observed differences between the May and June samplings should be interpreted cautiously and considered associations occurring in temporal proximity to the fire event rather than direct evidence of a causal relationship. Accordingly, the present work should be regarded as a pilot and exploratory study.

Despite these limitations, monitoring oxidative and epigenetic biomarkers in bees can provide valuable information on the sublethal effects of environmental stressors; however, the interpretation should remain strictly biomarker-specific and should not be generalized to systemic or regulatory-level biological effects based on the current dataset. Honeybees remain promising bioindicators due to their ecological relevance and shared exposure pathways with humans, offering potential insights for environmental monitoring and ecosystem health assessment, with broader relevance for One Health perspectives.

## Supplementary Information

Below is the link to the electronic supplementary material.

ESM 1 (DOCX 457 KB)

## Data Availability

All data are included in this article. Data will also be available on reasonable request.
