# Supplementary material for: Exploring oxidative stress and epigenetic biomarkers in bees: insights into health effects of air pollution and diet – a pilot study
Source: Environ Sci Pollut Res Int. 2026 Jul 7;33(21):10954–62. doi: 10.1007/s11356-026-38040-z (PMC13369631; doi:10.1007/s11356-026-38040-z)
Supplement: Supplementary file 1 — (DOCX 457 KB) [file 11356_2026_38040_MOESM1_ESM.docx]

Supplementary material

**Exploring oxidative stress and epigenetic biomarkers in bees: insights into health effects of air pollution and diet – a pilot study**

Roberta Giorgione^1^, Marcello Messi^1^, Daniela Pigini^2^, Maria Luisa Astolfi^1,3,^*

^1^ Department of Chemistry, Sapienza University of Rome, P.le Aldo Moro 5, 00185 Rome, Italy;

^2^ Department of Medicine, Epidemiology, Environmental and Occupational Hygiene, INAIL, via Fontana Candida 1, 00078 Monte Porzio Catone, Italy;

^3^ Research Center for Applied Sciences to the Safeguard of Environment and Cultural Heritage (CIABC), Sapienza University of Rome, P.le Aldo Moro 5, 00185 Rome, Italy

* Corresponding author. Tel: +39 0649913748, E-mail: [marialuisa.astolfi@uniroma1.it](mailto:marialuisa.astolfi@uniroma1.it)


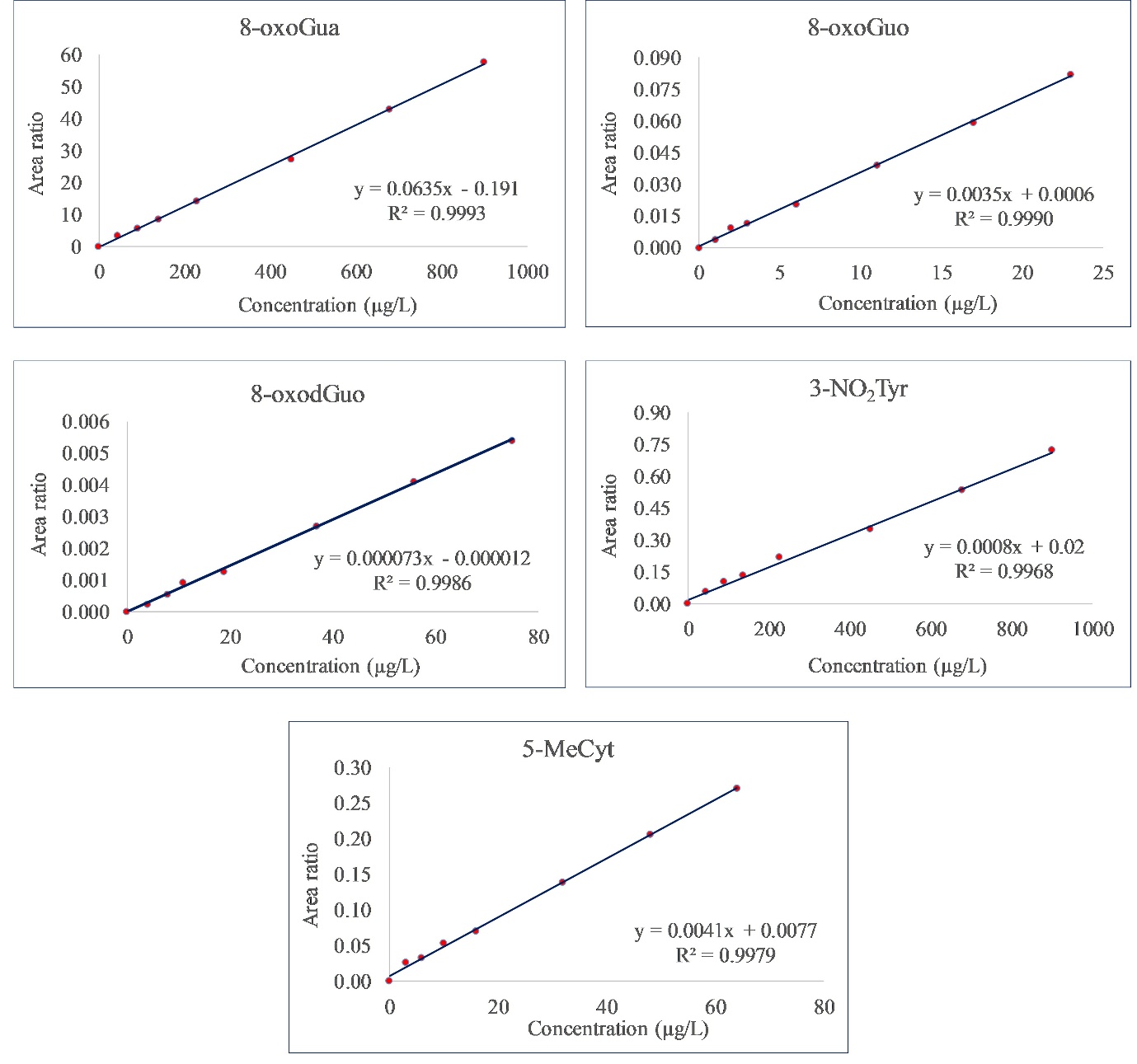


**Fig. S1** Calibration curves obtained for the HPLC‒MS/MS analysis of the target analytes


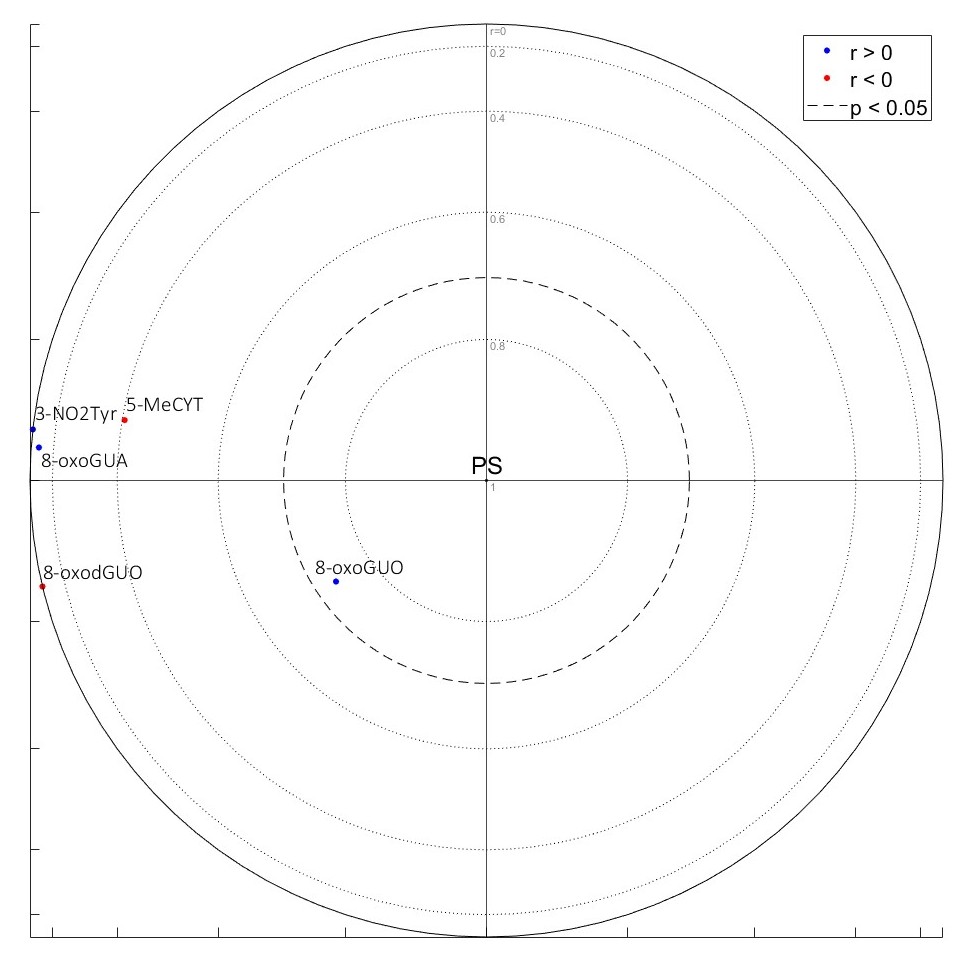


**Fig. S2** Focused Principal Component Analysis (FPCA) of physiological stress (PS) and oxidative stress biomarkers in honeybees. FPCA correlation circle showing the relationships between PS, determined as reported in Giampaoli et al. (2025), and the oxidative stress biomarkers measured in the present study. Blue dots indicate positive correlations (r > 0), red dots indicate negative correlations (r < 0). The dashed circle represents the significance threshold (p < 0.05). A significant positive correlation was observed between PS and 8-oxoGuo, suggesting a link between systemic stress and RNA oxidation


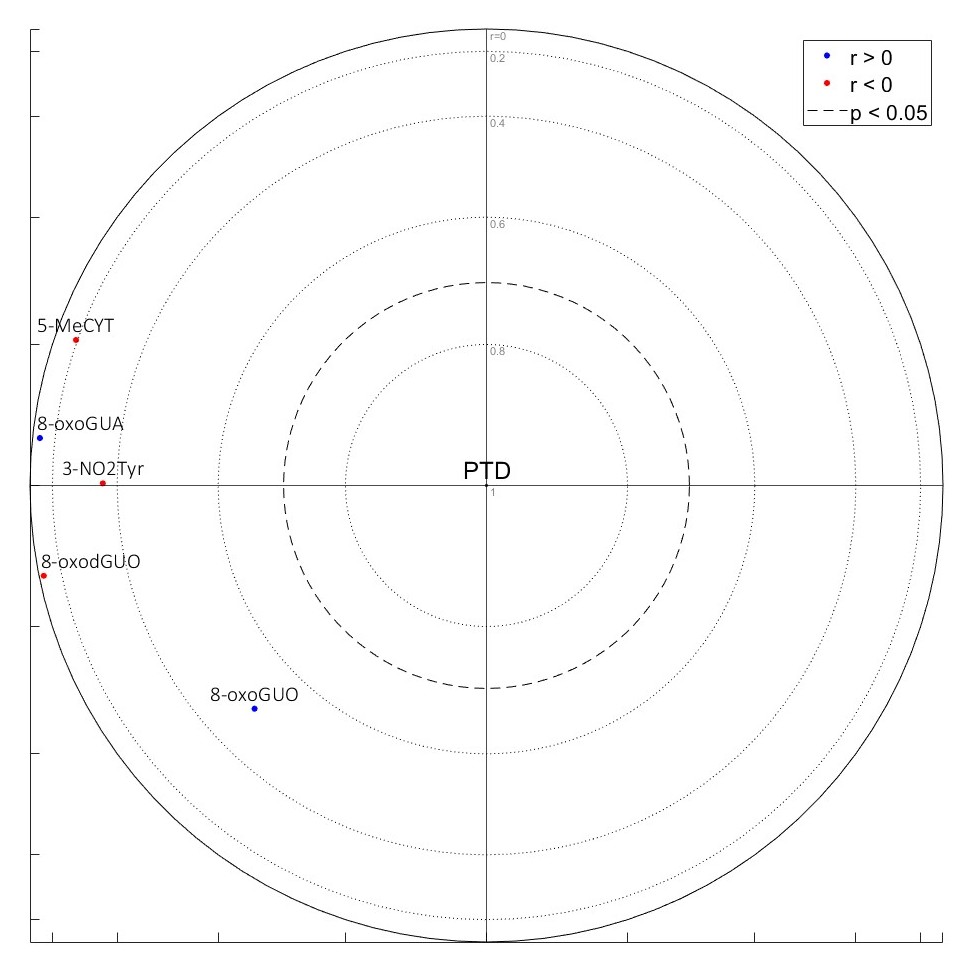


**Fig. S3** Focused Principal Component Analysis (FPCA) of potential post-transcriptional damage (PTD) and oxidative stress biomarkers in honeybees. FPCA correlation circle showing the relationships between PTD, as defined in Giampaoli et al. (2025), and the oxidative stress biomarkers analyzed in this study. Blue dots indicate positive correlations (r > 0), red dots indicate negative correlations (r < 0). The dashed circle represents the significance threshold (p < 0.05). No significant correlations were observed, suggesting that PTD and nucleic acid oxidation markers may reflect distinct phases of the cellular stress response

**Reference**

Giampaoli, O., Messi, M., Merlet, T., Sciubba, F., Canepari, S., Spagnoli, M., Astolfi, M.L. (2025) Landfill fire impact on bee health: beneficial effect of dietary supplementation with medicinal plants and probiotics in reducing oxidative stress and metal accumulation. Environ. Sci. Pollut. Res., 1-17
